# Menstrual and Reproductive Function Before and After Metabolic Bariatric Surgery

**DOI:** 10.1007/s11695-026-08852-6

**Published:** 2026-08-05

**Authors:** Martin Whyte, Stephanie Attersley-Smith, Kathryn Hart, Sophia Stone, Christopher Pring, Jill Shawe

**Affiliations:** 1 University of Surrey, Guildford, UK; 2 University Hospitals Sussex NHS Foundation Trust, Chichester, UK; 3 Royal Cornwall Hospital Trust, Truro, UK

## Abstract

**Aims:**

To evaluate the impact of metabolic bariatric surgery on menstrual function, hormonal profiles, and reproductive health in women of reproductive age.

**Materials and methods:**

In this prospective cohort study, women aged 18–45 years undergoing Roux-en-Y gastric bypass or sleeve gastrectomy were recruited from a UK bariatric centre and followed for up to 24 months. Control participants were women with obesity receiving non-surgical weight management.

Participants consented to clinical, biochemical, ultrasonographic, and questionnaire-based assessments of menstruation and pregnancy intentions.

**Results:**

Eighty-four women underwent surgery (49 bypass, 35 sleeve); 18 controls without surgery. By 24 months, 15 were lost to follow-up; mean excess weight loss was 64–73% with no difference between procedures. Anti-Mullerian Hormone (AMH) (n = 33) decreased post-surgery (16.6 to 16.0 pmol/L; P < 0.001); sex hormone-binding globulin (SHBG) increased (30.6 to 66.8 nmol/L; P < 0.001), and free androgen index (FAI) decreased (3.92 to 1.84; P < 0.001). Irregular menstruation and hirsutism decreased. Among 66 women with baseline transvaginal ultrasound scans, 33% had polycystic ovarian morphology, reducing to 10% postoperatively (n = 17); ovarian volume decreased significantly. At baseline (n = 76), 31% of surgical participants expressed a desire for pregnancy. Twenty pregnancies occurred, most 12–24 months post-surgery.

**Conclusions:**

Metabolic bariatric surgery was associated with > 30 kg weight loss by 24 months, reduced FAI, and improvements in menstrual regularity, and ovarian morphology, suggesting potential improvement in fertility-related parameters. Given high pregnancy intention, findings support the need for integrated reproductive counselling and preconception care within bariatric pathways.

**Supplementary Information:**

The online version contains supplementary material available at https://doi.org/10.1007/s11695-026-08852-6.

## Key points

Metabolic bariatric surgery is increasingly being carried out in women of reproductive age, often specifically to address fertility issues associated with obesity.

We identified a desire for pregnancy as being a motivator for metabolic bariatric surgery in this younger, female population opportunity for preconception care.

After surgery, improvement was found in menstrual cycle frequency and regularity, hirsutism, and resolution of polycystic ovaries on ultrasound.

We advocate for clinicians to consider menstrual dysfunction and impaired fertility as a driver for medical intervention for obesity.

**Supplementary Information:**

The online version contains supplementary material available at https://doi.org/10.1007/s11695-026-08852-6.

## Introduction

Women experience a higher prevalence of obesity than men [1], with 57% of women in the UK classified as overweight (BMI > 25 kg/m^2^) and 27% living with obesity (BMI > 30 kg/m^2^) [1]. Women of reproductive age comprise a large proportion of those undergoing metabolic bariatric surgery (MBS). In the UK, 77% of procedures in 2018 were performed in women, nearly half under 45 years [2]. In the Longitudinal Assessment of Bariatric Surgery 2 Study (LABS2), almost half of women aged 18–44 undergoing surgery were planning for pregnancy afterwards [3]. 

Polycystic ovarian syndrome (PCOS) affects 5–10% of women, with over half living with obesity [4]. PCOS is the leading cause of anovulatory subfertility and is associated with increased miscarriage risk [5]. Obesity independently impairs fertility and conception, even without PCOS [6], and decreases the likelihood of a live birth [7], likely through impaired oocyte quality and endometrial receptivity [7, 8]. Dietary weight loss can reduce ovarian volume and follicle count, restoring ovulation in women with PCOS and obesity [6, 9]. A systematic review showed that ovulation improves with weight loss of < 16% in women with anovulatory cycles due to PCOS [10]. 

Pregnancy following MBS is generally safer for both mother and fetus than pregnancy with obesity [11–13]. However, post-surgical nutritional deficiencies raise concerns about fetal growth restriction and small-for-gestational-age [12–14]. Consequently, the ideal timing and management of pregnancy after MBS remains unclear [12–14]. Given the high number of women of reproductive age undergoing surgery and the desire for pregnancy afterward, it is important to prospectively evaluate the impact of MBS on reproductive function. We investigated menstrual and reproductive function in women aged 18–45 years undergoing MBS.

## Methods

### Participants

Women were prospectively recruited from a UK bariatric centre prior to surgery and followed for up to two years. In the UK, obesity services are organised into tiers: Tier 1 - public health/lifestyle advice, Tier 2 - structured community weight management programmes, and Tier 3 - specialist multidisciplinary medical intervention. Control participants were recruited from the same Tier 3 services. Inclusion criteria were females aged 18–45 years (defined hereafter as women of reproductive age), meeting National Institute for Health and Care Excellence (NICE) criteria for surgery (BMI ≥ 35 kg/m² with ≥ 1 obesity-related comorbidity, or > 40 kg/m² without comorbidity), and listed for surgery by an NHS multidisciplinary team - awaiting gastric bypass or sleeve gastrectomy. Exclusion criteria included prior weight loss surgery (except gastric band) and current pregnancy at consent. Gastric banding was permitted as it is a reversible, primarily restrictive procedure with limited long-term metabolic and hormonal effects compared with MBS [15, 16].

### Design

**Fig. 1:**
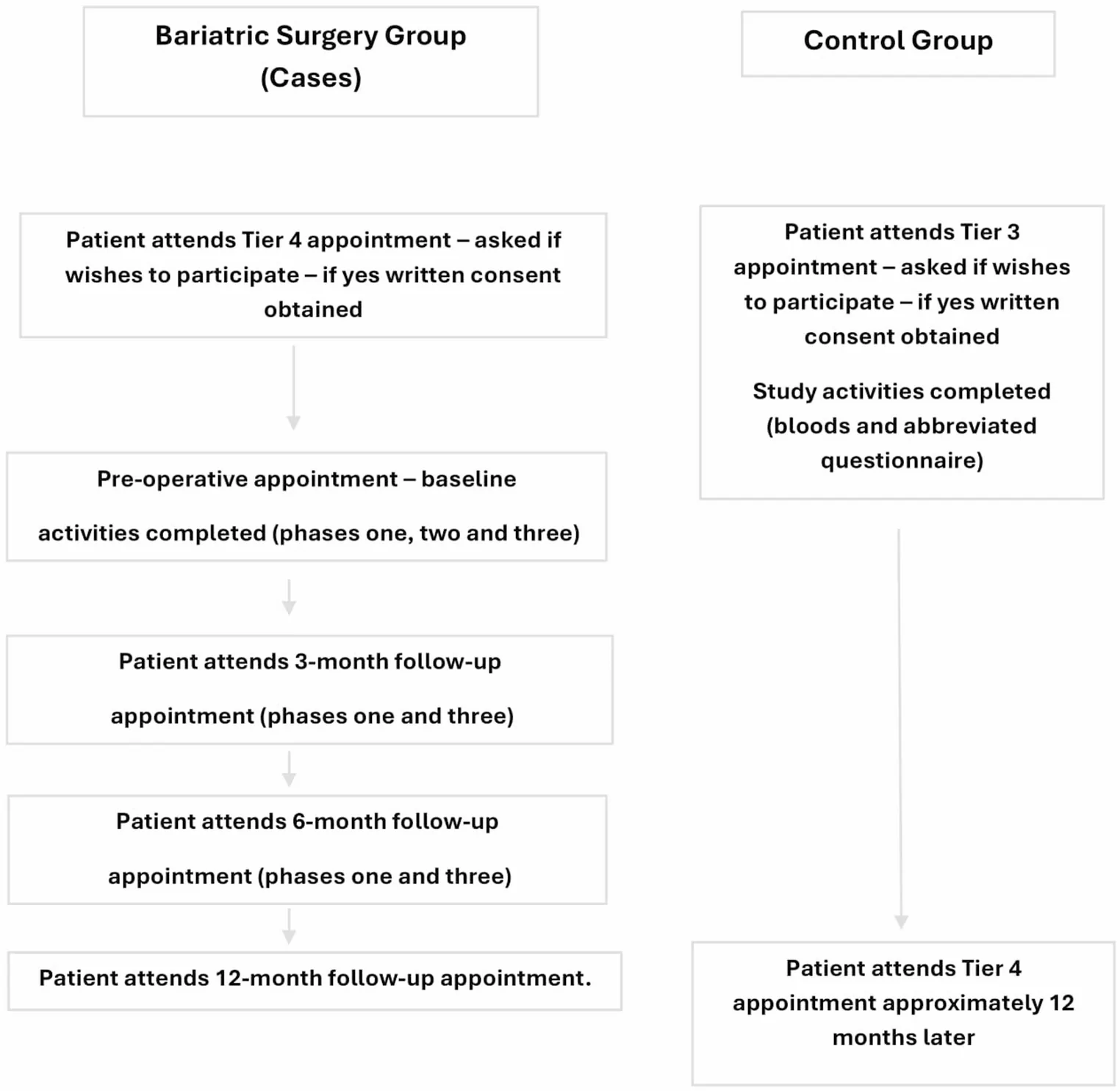
Participant flow through study

An observational, prospective cohort study investigating reproductive health outcomes in women of reproductive age undergoing MBS. Figure 1 outlines the study protocol for both groups.

### Procedure

i) Surgical cases

The study comprised three interlinked phases. 1: Medical record linkage; 2: Endocrine and gonadal assessment (biochemistry and imaging pre-surgery and 12–24 months post operatively). 3: Patient reported outcomes - Reproductive Health after Metabolic Surgery Study (RHAMS) questionnaire, including socioeconomic status (SES), reproductive health, nutrition and quality of life (QoL). All participants completed phase one, with optional enrolment in phases two and three.

ii) Control group

Controls underwent body weight and plasma anti-mullerian hormone (AMH) assessment and completed questionnaires at baseline and at 12–20 months. A contemporaneous control cohort was included as a contextual reference for temporal variability; the primary inferential analyses were prespecified as within-person longitudinal changes in the surgical cohort. Where exact alignment with surgical follow-up was not possible, assessments were conducted within an 8-month window, reflecting service constraints during COVID-19.

### Sample Size

Based on serum AMH change and standard deviation from Chiofalo et al. [17], a sample size of 75 provided 80% power to detect a 0.48 ng/ml within person change in AMH - considered the limit of physiological AMH fluctuation in a menstrual cycle [18–20]. 

### Materials

#### Phase 1. Medical Record linkage

At baseline, medical records (via SemaHelix, ClinicYou, Evolve, PACS) were reviewed for demographics and anthropometric data. For cases, records were reviewed at 3, 6, 12, and 24 months post-op. For controls, at 12 months.

#### Phase 2. Endocrine and Gonadal Assessment

Blood for total testosterone, sex hormone-binding globulin (SHBG), and AMH were taken at baseline and 12 months. AMH analysed using chemiluminescent immunoassay (Roche Cobas e801), testosterone via mass spectrometry, and SHBG using Siemens Advia Centaur assay. Free Androgen Index (FAI) calculated as total testosterone/SHBG × 100. Transvaginal scans were performed at baseline and 12 months by a gynaecologist using Toshiba Aplio 300 or Samsung Hera W10. Polycystic ovarian morphology was diagnosed if > 12 antral follicles were seen, or ovarian volume > 10 ml [21].

#### Phase 3. Patient Reported Outcomes

Participants completed a 14-item reproductive health questionnaire at baseline, 3-, 6-, and 12-months post-surgery. Androgen excess was self-assessed using the Ferriman-Gallwey scale [22].

### Statistical Analysis

The study was designed with a single primary endpoint (within-person change in AMH following MBS), for which the sample size calculation was performed. Longitudinal changes in weight, SHBG, testosterone, FAI, AMH, and ovarian volume were analysed using a linear mixed-effects model, selected a priori to allow for inclusion of all available repeated measures and account for within-subject correlation under a missing-at-random assumption. No imputation was performed; missing data were handled within the mixed-effects framework, which uses all available observations. Spearman’s correlations assessed associations between biochemical/ultrasound markers and weight metrics at baseline and follow-up. Analyses were performed using SPSS (v28), with *P* < 0.05 considered significant. Between-group comparisons were considered secondary and exploratory, and interpretation focuses on effect size and direction rather than formal hypothesis testing. No formal correction for multiple testing was applied.

### Ethical Review

Favourable ethical opinion was granted by the West of Scotland Research Ethics Service, 4th December 2018 (ref: 18/WS/0049).

## Results

A total of *n* = 99 patients had baseline data collected (Table 1), of which *n* = 84 went on to have MBS (*n* = 49 (58.3%) Roux-en-Y gastric bypass, *n* = 35 (41.7%) gastric sleeve). The remaining fifteen women either did not have surgery in the study period (*n* = 12) or withdrew (*n* = 3). Eighteen females attending Tier 3 services were recruited as controls.

**Table 1:** Baseline characteristics of women with obesity recruited to a case-control study of metabolic bariatric surgery

	Cases Mean ± SD			Controls Mean ± SD (*n* = 18)	*P* ^1^	*P* ^2^
	All cases (*n* = 99)	Sleeve (*n* = 35)	RYGB (*n* = 49)			
Baseline weight (kg)	135.7 ± 23.3	127.8 ± 20.5	138.3 ± 21.6	144.5 ± 32.0	0.170	0.027
Baseline BMI (kg/m^2^)	49.5 ± 8.8	46.8 ± 7.4	49.7 ± 8.1	52.7 ± 9.7	0.099	0.111
Baseline excess weight (kg)	66.2 ± 22.6	59.4 ± 19.9	68.5 ± 21.1	76.2 ± 29.4	0.104	0.048
Age at recruitment (years)	33.8 ± 5.8	34.3 ± 6.2	33.9 ± 5.2	32.8 ± 7.1	0.470	0.697
BMI category N (%)						
BMI 25–30 kg/m^2^	1 (1.0%)	1 (2.9%)	0 (0%)	0 (0.0%)		
BMI > 30–40 kg/m^2^	7 (7.1%)	4 (11.4%)	2 (4.1%)	0 (0.0%)		
BMI > 40 kg/m^2^	91 (91.9%)	30 (85.7%)	47 (95.9%)	18 (100.0%)		

^1^independent t-test, cases versus controls; ^2^independent t-test, sleeve versus RYGB

RYGB: roux en Y gastric bypass

### Phase 1

#### Baseline Characteristics

Table 1 shows baseline characteristics for cases (*n* = 99), controls (*n* = 18), and by surgical subgroup. There were no baseline differences between cases and controls. Patients undergoing RYGB were heavier than those having sleeve gastrectomy. Most surgical cases and controls were of White ethnicity (86.8% and 77.8%, respectively). No significant differences were observed in deprivation decile, education, employment, relationship status, smoking, or alcohol use (Appendix Table 1). Follow-up data completeness varied by outcome, reflecting participant choice and service constraints; denominators are reported as appropriate.

#### Weight Loss by Surgical Procedure

Baseline weight was 127.8 ± 20.5 kg (BMI 46.8 ± 7.4 kg/m^2^) in the gastric sleeve group and 138.3 ± 21.6 kg (BMI 49.7 ± 8.1 kg/m^2^) in the RYBG group (*P* = 0.027). Total weight decreased from baseline to 24 months (*P* < 0.001). By 24-months, weights were 91.9 ± 22.0 kg and 88.6 ± 24.6 kg, respectively. There was a significant interaction between time and operation (*P* = 0.002), indicating that the magnitude of weight loss differed by operative type. Mean excess weight loss (EWL) at 6 months was 58.4 ± 22.3% in the sleeve group and 63.8 ± 20.4% in the RYGB group. At 12 months, EWL was 69.6 ± 32.3% and 76.9 ± 25.3% in the sleeve and RYGB groups, respectively. At 24 months, mean EWL was 66.0 ± 32.4% in the sleeve group and 77.2 ± 27.9% in the RYGB group. A linear mixed-effects model showed a significant effect of time, with EWL higher at 12 months (+ 10.7%, *p* < 0.001) and 24 months (+ 7.1%, *P* = 0.005) compared with 6 months. There was no evidence of a time × procedure interaction (χ² [2] = 1.77, *P* = 0.413), indicating that EWL trajectory did not differ significantly between sleeve and RYGB groups.

### Phase 2

#### AMH and Testosterone

Following surgery, AMH decreased from 16.6 ± 15.6 pmol/L to 16.0 ± 19 pmol/L, *P* < 0.001). In the control group, AMH was unchanged (13.3 ± 11.9 to 15.2 ± 9.9; *P* = 0.374). However, the change in AMH was not different between cases and controls (*P* = 0.396).

**Fig. 2:**
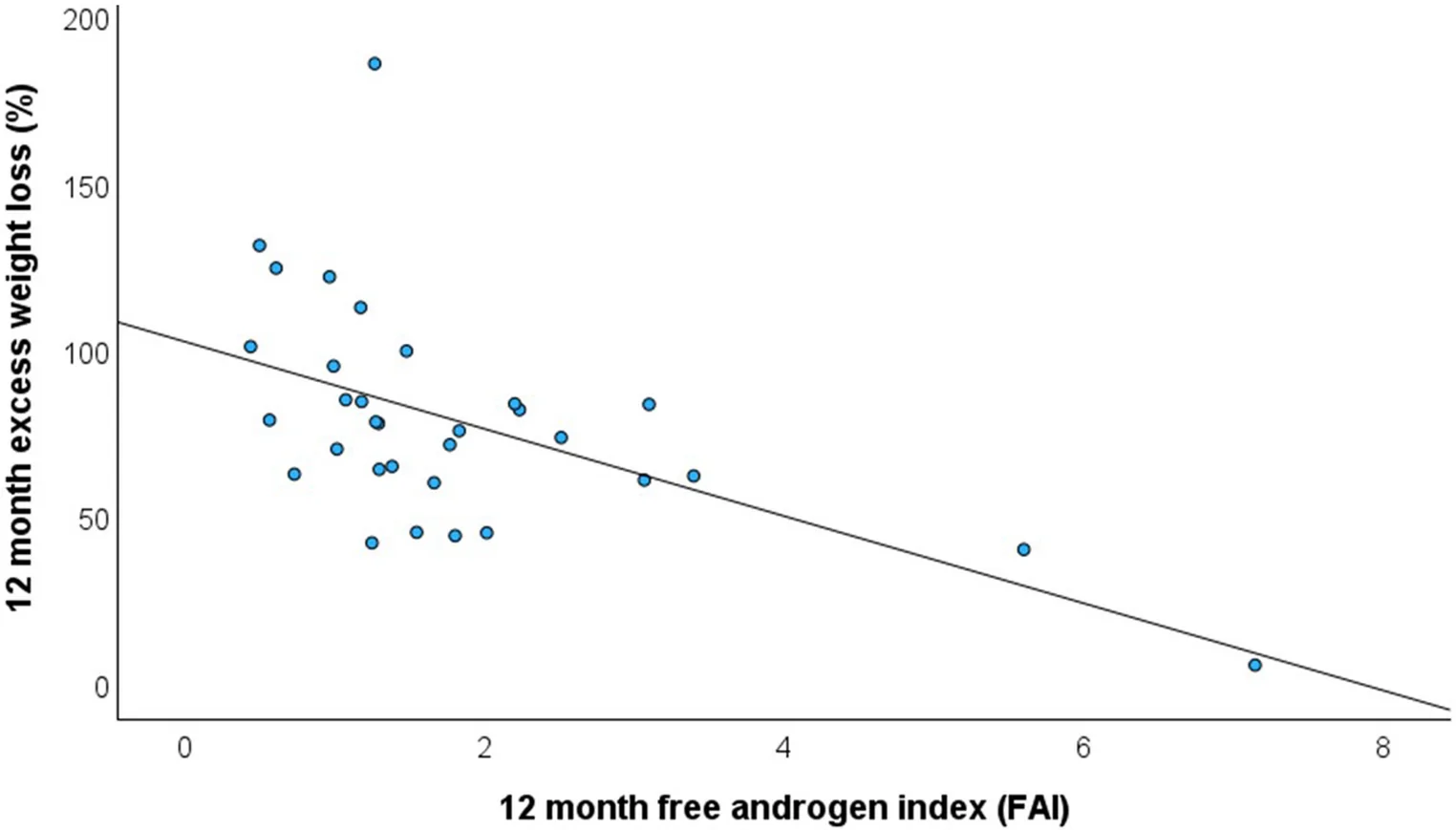
Association between Free Androgen Index (FAI) and percentage excess weight loss at 12 months in women following metabolic bariatric surgery

Total testosterone was unchanged after 12 months (0.96 ± 0.42 to 0.95 ± 0.35; *P* = 0.895). SHBG increased from 30.6 ± 14.5 nmol/L to 66.8 ± 33.5 nmol/L at 12-months post-surgery (*P* < 0.001) and correlated with percentage EWL (Rho 0.367; *P* = 0.001). FAI decreased from 3.92 ± 2.88 to 1.84 ± 1.4 (*P* < 0.001) and, at 12-months, negatively correlated with percentage EWL (*r*=-0.135, *P* < 0.001; Fig. 2).

#### Polycystic Ovaries

Of 76 women having a baseline transvaginal USS, *n* = 25 (32.9%) had polycystic ovarian morphology. Twenty women had repeat USS 12–24 months after surgery. Baseline BMI 46.5 ± 7.1 vs. 49.9 ± 8.3 kg/m^2^ (*P* = 0.111), and age (33.5 ± 5.8 vs. 33.7 ± 5.1 years, *P* = 0.903) were no different between those who did and did not have follow up USS. Of the 20 women undergoing repeat ovarian USS 12–24 months after surgery, two (10.0% of those imaged) had polycystic ovarian morphology. Ovarian volume reduced from 7.4 ± 3.9 (*n* = 76) to 5.7 ± 2.6 (*n* = 20), 12–24 months after surgery (*P* = 0.005) but was only significant after gastric sleeve (-2.4 ml, *P* = 0.018) and not RYGB − 1.7mls, *p* = 0.091).

### Phase 3

#### Contraception and Pregnancy Intention

Family planning was self-reported via the RHAMS questionnaire. Baseline data were missing for 20.0% of cases. Among respondents, 43.0% of cases and 22.2% of controls were not using contraception. Among contraceptive users, 46.7% of cases and 42.8% of controls used long-acting reversible methods (injection, intrauterine device or implant); while 24.4% and 14.2% used condoms, respectively. No participants used combined hormonal contraceptives; 22.2% of cases and 42.9% of controls used progestogen only pills.

Approximately half of women in both groups had at least one child and 31.3% of cases and 44.4% of controls expressed an intent to conceive. Nearly 10% of both groups had experienced sub-fertility, and one-quarter had a pre-existing PCOS.

#### Clinical Hyperandrogenism

Ferriman-Galwey score > 8 was reported in *n* = 22 (26.2%) of cases at baseline, falling to *n* = 5 (6.0%) at 12-months.

#### Menstrual Regularity

The number of women with self-reported irregular periods reduced over follow-up (Table 2), persisting after excluding women using hormonal contraception. The median number of self-reported periods per month tended to increase throughout follow-up, particularly amongst those with PCOS at baseline (Table 2).

**Table 2:** Self-reported menstrual cycle characteristics for women undergoing metabolic bariatric surgery, pre-and post-surgery (*n* = 99)

N (% of all *n* = 99 participants)	Baseline	3 months	6 months	12 months	24 months
All women					
Irregular or absent periods	49 (49/*49.5%*)	23 (23/*23.2%*)	14 (14/*14.1%*)	13 (13/*13.1%*)	5 (5/*5*.*1%*)
Regular periods (all)	30 (30/*30.3%*)	21 (21/21.2*%*)	21 (21/*21.2%*)	21 (21/*21.2%*)	21 (21/*21.2%*)
Unknown	20 (20.2%)	55 (55.6%)	64 (64.6%)	65 (65.7%)	73 (73.7%)
No hormonal contraception at baseline (*n* = 28)					
Irregular periods	15 (15/*15.2%*)	14 (14/*14.1%)*	8 (8/*8.1%*)	7 (7/*7.1%*)	4 (4/*4.0%*)
Regular periods	13 (13/*13.1%*)	12 (12/*12.1%*)	14 (14/*14.1%*)	13 (13/*13.1%*)	17 (17/*17.2%*)
Missing data	0	2 (2.0%)	6 (6.0%)	8 (8.1%)	7 (7.1%)
Numbers with increased frequency/regularity of periods since baseline	N/A	12 (12/*12.1%)*	19 (19/*19.2%*)	16 (16/*16.2%*)	12 (12/*12.1%*)
Periods per month Median (25%, 75%)					
All cases	0.17 (0.00,0.75) (*n* = 32)	0.67 (0.00, 1.00) (*n* = 30)	0.67 (0.17, 1.00) (*n* = 25)	1.00 (0.00, 1.00) (*n* = 24)	1.00 (0.33, 1.00) (*n* = 17)
PCOS at baseline	0.00 (0.00, 0.50) (*n* = 14)	0.67 (0.17, 1.00) (*n* = 13)	0.83 (0.42, 1.00) (*n* = 8)	1.00 (0.33, 1.00) (*n* = 9)	1.00 (0.33, 1.00) (*n* = 5)
Non-PCOS at baseline	0.625 (0.125, 0.854) (*n* = 18)	0.33 (0.00, 1.00) (*n* = 17)	0.67 (0.00, 1.00) (*n* = 17)	0.67 (0.00, 1.00) (*n* = 15)	1.00 (0.17, 1.00) (*n* = 12)

#### Diagnosis of PCOS

Thirty-five women (from *n* = 99) of the case group (35.9%) met the Rotterdam Criteria for PCOS at baseline; *n* = 7 (20.0% of diagnoses) due to a combination of oligo/amenorrhoea and PCO; *n* = 14 (40.0%) due to a combination of oligo/amenorrhoea and hirsutism and *n* = 2 (5.7%) due to PCO and hirsutism. The remaining 12 (34.3%) met all three diagnostic criteria. Ten women with baseline PCOS self-reported normalization of periods by 12 months. Mean EWL at resumption of a regular cycle was 53.7 ± 20.0%. Two women reported resolution of clinical hirsutism by 12 months; their EWLs at resolution were 31.0% and 54.0%.

#### Pregnancies

A total of 20 pregnancies were reported from the surgical group during study follow-up, 17 resulting in a live birth and three ending in first trimester miscarriages. The earliest pregnancy was conceived three months post-operatively, with most conceived between 12 and 24 months after surgery.

## Discussion

We investigated menstrual and reproductive function in women undergoing MBS. By 24 months, EWL was 64–73%, with similar trajectories between sleeve gastrectomy and Roux-en-Y gastric bypass. This substantial weight loss was accompanied by improvements in androgen status, menstrual regularity, and ovarian morphology, suggesting improved fertility potential after surgery. The Lancet Commission on obesity highlighted that BMI is insufficient to capture obesity-related health impacts, and anovulation and PCO were recognised as clinical consequences [23]. We advocate that clinicians recognise menstrual dysfunction and infertility as indications for obesity treatment, that this period is a window for preconception care, and that family planning be emphasised to prevent unintended pregnancies.

AMH is considered a marker of reproductive potential [24]. The magnitude of AMH change was small and likely within physiological variability and therefore its clinical significance remains uncertain. AMH helps predict ovarian response to gonadotrophin stimulation with IVF, with approximately ≤ 6 pmol/l indicating low response and ≥ 14–25 pmol/l indicating high response [25, 26]. Although PCOS is associated with higher AMH levels, this does not equate to improved fertility [27], and a reduction in AMH may paradoxically signal improvement in the syndrome [27]. The clinical significance of AMH changes remains uncertain, with conflicting views on its reliability as a predictor of ovarian reserve in obesity [28–30]. 

Weight loss was likely responsible for improved surrogates of reproductive outcomes, rather than a direct surgical effect as randomized control trial data show that a very low calorie diet, resulting in mean 6.6 kg weight loss, improved outcomes in women with obesity undergoing fertility treatment [31]; less weight loss (mean 4.6 kg) didn’t appear to benefit assisted conception [32]. Approximately 5% weight loss may restore ovulation in women with BMI > 30 kg/m^2^ [33–35]. 

Our study included women with and without PCO. The observed menstrual improvements align with observational studies of PCO [36–41] and the BAMBINI trial (bariatric surgery vs. medical care for obesity and polycystic ovarian syndrome related infertility). This found that women undergoing MBS had 2.5 times more spontaneous ovulations than those medically treated [42]. In our cohort, 20 pregnancies were identified: 17 resulted in a live birth and 3 ended in first trimester miscarriage. Importantly, our study was not designed to assess fertility rates or timing of conception. Small case control studies have reported increased [43], unchanged [44], or reduced [45] miscarriage rates after MBS. Despite concerns about early post-op pregnancies, no difference in birth weight was observed between babies conceived within, or after, the first year [46]. Although this study was not testing optimal timing of reproductive counselling, our findings highlight that fertility potential may improve soon after surgery. We suggest integrated reproductive counselling and preconception care be incorporated at surgical referral and revisited during postoperative follow-up [13, 47]. 

### Strengths and Limitations

Strengths include a representative cohort of women of reproductive age undergoing MBS, with healthcare record linkage, biochemical, ultrasound, and qualitative data to assess the impact of MBS on menstrual and reproductive function.

Limitations include exclusion of patients with prior weight-loss surgery (except gastric band), which may limit applicability to populations where revision surgeries are common. Exclusion of pregnant women from consent may bias toward women with existing fertility challenges. The control cohort was smaller and not fully temporally aligned with surgical follow-up. This reflected the pragmatic design of the study within NHS pathways, particularly during the COVID-19 pandemic. Single-centre NHS recruitment may limit generalisability, and pregnancy numbers were too few to draw conclusions about conception safety post-surgery.

Longitudinal studies are needed to track menstrual regularity and reproductive outcomes over several years [48–51]. Research is needed to identify the optimal conception timing post-surgery as existing studies are often small, retrospective, and do not reliably record miscarriage data [45, 52, 53]. 

In summary, we have shown in a prospective cohort study of women with and without PCOS, that MBS led to biochemical, clinical and ovarian ultrasonographic changes that could improve the ability to conceive.

## Supplementary Information

Below is the link to the electronic supplementary material.

Supplementary Material 1 (DOCX 21.7 KB)

Supplementary Material 2 (DOCX 132 KB)

Supplementary Material 3 (DOCX 158 KB)

## Data Availability

The data that support the findings of this study contain anonymised patient information and are not publicly available due to confidentiality restrictions. Data may be made available from the corresponding author upon reasonable request and subject to appropriate data sharing agreements.

## References

[1] Statistics on Obesity. Physical Activity and Diet - England, 2018 [PAS]. In: Statistics OoN, editor.

[2] The United Kingdom National Bariatric Surgery Registry. Third Registry Report 2020. The British Obesity and Metabolic Surgery Society.

[3] Gosman GG, King WC, Schrope B, Steffen KJ, Strain GW, Courcoulas AP, et al. Reproductive health of women electing bariatric surgery. Fertil Steril. 2010;94(4):1426–31. PubMed PMID: 19815190. Pubmed Central PMCID: PMC2888936. Epub 2009/10/10. 19815190 10.1016/j.fertnstert.2009.08.028 PMC2888936

[4] Bhide P, Homburg R. Anti-Mullerian hormone and polycystic ovary syndrome. Best Pract Res Clin Obstet Gynaecol. 2016;37:38–45. PubMed PMID: 27103234. Epub 2016/04/23. 27103234 10.1016/j.bpobgyn.2016.03.004

[5] Wartena R, Matjila M. Polycystic ovary syndrome and recurrent pregnancy loss, a review of literature. Front Endocrinol (Lausanne). 2023;14:1183060. PubMed PMID: 38027110. Pubmed Central PMCID: PMC10643146. Epub 2023/11/29. 38027110 10.3389/fendo.2023.1183060 PMC10643146

[6] Medenica S, Spoltore ME, Ormazabal P, Marina LV, Sojat AS, Faggiano A, et al. Female infertility in the era of obesity: The clash of two pandemics or inevitable consequence? Clin Endocrinol (Oxf). 2023;98(2):141–52. PubMed PMID: 35644933. Pubmed Central PMCID: PMC10084349. Epub 2022/06/02. 35644933 10.1111/cen.14785 PMC10084349

[7] Cavalcante MB, Sarno M, Peixoto AB, Araujo Junior E, Barini R. Obesity and recurrent miscarriage: A systematic review and meta-analysis. J Obstet Gynaecol Res. 2019;45(1):30–8. PubMed PMID: 30156037. Epub 2018/08/30. 30156037 10.1111/jog.13799

[8] Broughton DE, Moley KH. Obesity and female infertility: potential mediators of obesity’s impact. Fertil Steril. 2017;107(4):840–7. PubMed PMID: 28292619. Epub 2017/03/16. 28292619 10.1016/j.fertnstert.2017.01.017

[9] Crosignani PG, Colombo M, Vegetti W, Somigliana E, Gessati A, Ragni G. Overweight and obese anovulatory patients with polycystic ovaries: parallel improvements in anthropometric indices, ovarian physiology and fertility rate induced by diet. Hum Reprod. 2003;18(9):1928–32. PubMed PMID: 12923151. Epub 2003/08/19. 12923151 10.1093/humrep/deg367

[10] Jarrett BY, Lujan ME. Impact of hypocaloric dietary intervention on ovulation in obese women with PCOS. Reproduction. 2017; 153(1): R15-R27. PubMed PMID: 27799625. Pubmed Central PMCID: PMC5411334. Epub 2016/11/02. 10.1530/REP-16-0385 PMC5411334 27799625

[11] Galazis N, Docheva N, Simillis C, Nicolaides KH. Maternal and neonatal outcomes in women undergoing bariatric surgery: a systematic review and meta-analysis. Eur J Obstet Gynecol Reprod Biol. 2014;181:45–53. PubMed PMID: 25126981. Epub 2014/08/16. 25126981 10.1016/j.ejogrb.2014.07.015

[12] Kwong W, Tomlinson G, Feig DS. Maternal and neonatal outcomes after bariatric surgery; a systematic review and meta-analysis: do the benefits outweigh the risks? Am J Obstet Gynecol. 2018;218(6):573–80. PubMed PMID: 29454871. Epub 2018/02/20. 29454871 10.1016/j.ajog.2018.02.003

[13] Shawe J, Ceulemans D, Akhter Z, Neff K, Hart K, Heslehurst N, et al. Pregnancy after bariatric surgery: Consensus recommendations for periconception, antenatal and postnatal care. Obes Rev. 2019;20(11):1507–22. PubMed PMID: 31419378. Pubmed Central PMCID: PMC6852078. Epub 2019/08/17. 31419378 10.1111/obr.12927 PMC6852078

[14] Savastano G, Caruso G, Pompeo D, Lobozzo B, Perrone G, Pecorini F, et al. Pregnancy and post-partum outcomes of obese women after bariatric surgery: A case-control study. Eur J Obstet Gynecol Reprod Biol. 2022;272:43–7. PubMed PMID: 35279640. Epub 2022/03/14. 35279640 10.1016/j.ejogrb.2022.03.016

[15] Arakawa R, Febres G, Cheng B, Krikhely A, Bessler M, Korner J. Prospective study of gut hormone and metabolic changes after laparoscopic sleeve gastrectomy and Roux-en-Y gastric bypass. PLoS ONE. 2020;15(7):e0236133. PubMed PMID: 32687546. Pubmed Central PMCID: PMC7371190. Epub 20200720. 32687546 10.1371/journal.pone.0236133 PMC7371190

[16] Holter MM, Dutia R, Stano SM, Prigeon RL, Homel P, McGinty JJ Jr., et al. Glucose Metabolism After Gastric Banding and Gastric Bypass in Individuals With Type 2 Diabetes: Weight Loss Effect. Diabetes Care. 2017;40(1):7–15. PubMed PMID: 27999001. Pubmed Central PMCID: PMC5180462. Epub 20161108. 27999001 10.2337/dc16-1376 PMC5180462

[17] Chiofalo F, Ciuoli C, Formichi C, Selmi F, Forleo R, Neri O, et al. Bariatric Surgery Reduces Serum Anti-mullerian Hormone Levels in Obese Women With and Without Polycystic Ovarian Syndrome. Obes Surg. 2017;27(7):1750–4. PubMed PMID: 28378209. Epub 2017/04/06. 28378209 10.1007/s11695-016-2528-y

[18] Lahlou N, Chabbert-Buffet N, Gainer E, Roger M, Bouchard P, VA2914 Study Group. Diphasic pattern of anti-mullerian hormone (AMH) in ovulatory cycles as evidenced by means of an ultra-sensitive assay: new insights into ovarian function. Fertil Steril, 2006;86(Suppl):p S11. 2007.

[19] Streuli I, Fraisse T, Chapron C, Bijaoui G, Bischof P, de Ziegler D. Clinical uses of anti-Mullerian hormone assays: pitfalls and promises. Fertil Steril. 2009;91(1):226–30. PubMed PMID: 18249391. Epub 2008/02/06. 18249391 10.1016/j.fertnstert.2007.10.067

[20] Wunder DM, Bersinger NA, Yared M, Kretschmer R, Birkhauser MH. Statistically significant changes of antimullerian hormone and inhibin levels during the physiologic menstrual cycle in reproductive age women. Fertil Steril. 2008;89(4):927–33. PubMed PMID: 17603052. Epub 2007/07/03. 17603052 10.1016/j.fertnstert.2007.04.054

[21] Balen AH, Laven JS, Tan SL, Dewailly D. Ultrasound assessment of the polycystic ovary: international consensus definitions. Hum Reprod Update. 2003;9(6):505–14. PubMed PMID: 14714587. Epub 2004/01/13. 14714587 10.1093/humupd/dmg044

[22] Ferriman D, Gallwey JD. Clinical assessment of body hair growth in women. J Clin Endocrinol Metab. 1961;21:1440–7. PubMed PMID: 13892577. Epub 1961/11/01. 13892577 10.1210/jcem-21-11-1440

[23] Rubino F, Cummings DE, Eckel RH, Cohen RV, Wilding JPH, Brown WA, et al. Definition and diagnostic criteria of clinical obesity. Lancet Diabetes Endocrinol. 2025;13(3):221–62. PubMed PMID: 39824205. Pubmed Central PMCID: PMC11870235. Epub 2025/01/18. 39824205 10.1016/S2213-8587(24)00316-4 PMC11870235

[24] La Marca A, Sighinolfi G, Radi D, Argento C, Baraldi E, Artenisio AC, et al. Anti-Mullerian hormone (AMH) as a predictive marker in assisted reproductive technology (ART). Hum Reprod Update. 2010;16(2):113–30. PubMed PMID: 19793843. Epub 2009/10/02. 19793843 10.1093/humupd/dmp036

[25] NICE. CG 156. Fertility problems: assessment and treatment. Published: 20 February 2013 Last updated: 06 September 2017.

[26] Bosch E, Labarta E, Zuzuarregui J, Iliodromiti S, Nelson SM. Prediction of ovarian response using the automated Elecsys anti-Mullerian hormone assay in gonadotrophin-releasing hormone antagonist cycles. Reprod Biomed Online. 2023;46(2):295–301. PubMed PMID: 36522281. Epub 20221101. 36522281 10.1016/j.rbmo.2022.10.012

[27] Komorowski AS, Hughes L, Sarkar P, Aaby DA, Kumar A, Kalra B, et al. Antimullerian hormone level predicts ovulation in women with polycystic ovary syndrome treated with clomiphene and metformin. Fertil Steril. 2024;121(4):660–8. PubMed PMID: 38154770. Pubmed Central PMCID: PMC10978249. Epub 2023/12/29. 38154770 10.1016/j.fertnstert.2023.12.031 PMC10978249

[28] Albu D, Albu A. The relationship between anti-Mullerian hormone serum level and body mass index in a large cohort of infertile patients. Endocrine. 2019;63(1):157–63. PubMed PMID: 30238328. Epub 2018/09/22. 30238328 10.1007/s12020-018-1756-4

[29] Bernardi LA, Carnethon MR, de Chavez PJ, Ikhena DE, Neff LM, Baird DD, et al. Relationship between obesity and anti-Mullerian hormone in reproductive-aged African American women. Obes (Silver Spring). 2017;25(1):229–35. PubMed PMID: 27925445. Pubmed Central PMCID: PMC5182136. Epub 2016/12/08. 10.1002/oby.21681 PMC5182136 27925445

[30] Lefebvre T, Dumont A, Pigny P, Dewailly D. Effect of obesity and its related metabolic factors on serum anti-Mullerian hormone concentrations in women with and without polycystic ovaries. Reprod Biomed Online. 2017;35(3):325–30. PubMed PMID: 28624344. Epub 2017/06/19. 28624344 10.1016/j.rbmo.2017.05.013

[31] Sim KA, Dezarnaulds GM, Denyer GS, Skilton MR, Caterson ID. Weight loss improves reproductive outcomes in obese women undergoing fertility treatment: a randomized controlled trial. Clin Obes. 2014;4(2):61–8. PubMed PMID: 25826729. Epub 2015/04/01. 25826729 10.1111/cob.12048

[32] Jeong HG, Cho S, Ryu KJ, Kim T, Park H. Effect of weight loss before in vitro fertilization in women with obesity or overweight and infertility: a systematic review and meta-analysis. Sci Rep. 2024;14(1):6153. PubMed PMID: 38486057. Pubmed Central PMCID: PMC10940611. Epub 2024/03/15. 38486057 10.1038/s41598-024-56818-4 PMC10940611

[33] Clark AM, Ledger W, Galletly C, Tomlinson L, Blaney F, Wang X, et al. Weight loss results in significant improvement in pregnancy and ovulation rates in anovulatory obese women. Hum Reprod. 1995;10(10):2705–12. PubMed PMID: 8567797. Epub 1995/10/01. 8567797 10.1093/oxfordjournals.humrep.a135772

[34] Price SA, Sumithran P, Prendergast LA, Nankervis AJ, Permezel M, Proietto J. Time to pregnancy after a prepregnancy very-low-energy diet program in women with obesity: substudy of a randomized controlled trial. Fertil Steril. 2020;114(6):1256–62. PubMed PMID: 33077241. Epub 2020/10/21. 33077241 10.1016/j.fertnstert.2020.06.033

[35] Rich-Edwards JW, Goldman MB, Willett WC, Hunter DJ, Stampfer MJ, Colditz GA, et al. Adolescent body mass index and infertility caused by ovulatory disorder. Am J Obstet Gynecol. 1994;171(1):171–7. PubMed PMID: 8030695. Epub 1994/07/01. 8030695 10.1016/0002-9378(94)90465-0

[36] Eid GM, Cottam DR, Velcu LM, Mattar SG, Korytkowski MT, Gosman G, et al. Effective treatment of polycystic ovarian syndrome with Roux-en-Y gastric bypass. Surg Obes Relat Dis. 2005;1(2):77–80. PubMed PMID: 16925218. Epub 2006/08/24. 16925218 10.1016/j.soard.2005.02.008

[37] Hu L, Ma L, Xia X, Ying T, Zhou M, Zou S, et al. Efficacy of Bariatric Surgery in the Treatment of Women With Obesity and Polycystic Ovary Syndrome. J Clin Endocrinol Metab. 2022;107(8):e3217–29. PubMed PMID: 35554540. Pubmed Central PMCID: PMC9282367. Epub 2022/05/14. 35554540 10.1210/clinem/dgac294 PMC9282367

[38] Jamal M, Gunay Y, Capper A, Eid A, Heitshusen D, Samuel I. Roux-en-Y gastric bypass ameliorates polycystic ovary syndrome and dramatically improves conception rates: a 9-year analysis. Surg Obes Relat Dis. 2012;8(4):440–4. PubMed PMID: 22169760. Epub 2011/12/16. 22169760 10.1016/j.soard.2011.09.022

[39] Lv B, Xing C, He B. Effects of bariatric surgery on the menstruation- and reproductive-related hormones of women with obesity without polycystic ovary syndrome: a systematic review and meta-analysis. Surg Obes Relat Dis. 2022;18(1):148–60. PubMed PMID: 34756568. Epub 2021/11/11. 34756568 10.1016/j.soard.2021.09.008

[40] Rozanska-Waledziak A, Bartnik P, Kacperczyk-Bartnik J, Czajkowski K, Waledziak M. The Impact of Bariatric Surgery on Menstrual Abnormalities-a Cross-Sectional Study. Obes Surg. 2020;30(11):4505–9. PubMed PMID: 32661954. Pubmed Central PMCID: PMC7524851. Epub 2020/07/15. 32661954 10.1007/s11695-020-04840-6 PMC7524851

[41] Turkmen S, Ahangari A, Backstrom T. Roux-en-Y Gastric Bypass Surgery in Patients with Polycystic Ovary Syndrome and Metabolic Syndrome. Obes Surg. 2016;26(1):111–8. PubMed PMID: 25975201. Epub 2015/05/16. 25975201 10.1007/s11695-015-1729-0

[42] Samarasinghe SNS, Leca B, Alabdulkader S, Dimitriadis GK, Davasgaium A, Thadani P, et al. Bariatric surgery for spontaneous ovulation in women living with polycystic ovary syndrome: the BAMBINI multicentre, open-label, randomised controlled trial. Lancet. 2024;403(10443):2489–503. PubMed PMID: 38782004. Epub 2024/05/24. 38782004 10.1016/S0140-6736(24)00538-5

[43] Deitel M, Stone E, Kassam HA, Wilk EJ, Sutherland DJ. Gynecologic-obstetric changes after loss of massive excess weight following bariatric surgery. J Am Coll Nutr. 1988;7(2):147–53. PubMed PMID: 3361039. Epub 1988/04/01. 3361039 10.1080/07315724.1988.10720232

[44] De Carolis S, Botta A, Del Sordo G, Guerrisi R, Salvi S, De Carolis MP, et al. Influence of Biliopancreatic Diversion on Pregnancy Outcomes in Comparison to Other Bariatric Surgery Procedures. Obes Surg. 2018;28(10):3284–92. PubMed PMID: 29909515. Epub 2018/06/18. 29909515 10.1007/s11695-018-3350-5

[45] Bilenka B, Ben-Shlomo I, Cozacov C, Gold CH, Zohar S. Fertility, miscarriage and pregnancy after vertical banded gastroplasty operation for morbid obesity. Acta Obstet Gynecol Scand. 1995;74(1):42–4. PubMed PMID: 7856431. Epub 1995/01/01. 7856431 10.3109/00016349509009942

[46] Dao T, Kuhn J, Ehmer D, Fisher T, McCarty T. Pregnancy outcomes after gastric-bypass surgery. Am J Surg. 2006;192(6):762–6. PubMed PMID: 17161090. Epub 2006/12/13. 17161090 10.1016/j.amjsurg.2006.08.041

[47] Cruz S, Matos A, Cruz S, Pereira S, Saboya C, Ramalho A. Pregnancy after 24 Postoperative Months of Roux-En-Y Gastric Bypass Presents Risk of Pregnancy Complications Similar to Pregnancy within the First Postoperative Year. Ann Nutr Metab. 2019;75(1):24–30. PubMed PMID: 31288227. Epub 20190709. 31288227 10.1159/000501423

[48] Jans G, Matthys C, Bel S, Ameye L, Lannoo M, Van der Schueren B, et al. AURORA: bariatric surgery registration in women of reproductive age - a multicenter prospective cohort study. BMC Pregnancy Childbirth. 2016;16(1):195. PubMed PMID: 27473473. Pubmed Central PMCID: PMC4966861. Epub 2016/07/31. 27473473 10.1186/s12884-016-0992-y PMC4966861

[49] Legro RS, Dodson WC, Gnatuk CL, Estes SJ, Kunselman AR, Meadows JW, et al. Effects of gastric bypass surgery on female reproductive function. J Clin Endocrinol Metab. 2012;97(12):4540–8. PubMed PMID: 23066115. Pubmed Central PMCID: PMC3513539. Epub 2012/10/16. 23066115 10.1210/jc.2012-2205 PMC3513539

[50] Luyssen J, Jans G, Bogaerts A, Ceulemans D, Matthys C, Van der Schueren B, et al. Contraception, Menstruation, and Sexuality after Bariatric Surgery: a Prospective Cohort Study. Obes Surg. 2018;28(5):1385–93. PubMed PMID: 29197048. Epub 2017/12/03. 29197048 10.1007/s11695-017-3033-7

[51] Rochester D, Jain A, Polotsky AJ, Polotsky H, Gibbs K, Isaac B, et al. Partial recovery of luteal function after bariatric surgery in obese women. Fertil Steril. 2009;92(4):1410–5. PubMed PMID: 18829008. Pubmed Central PMCID: PMC2818247. Epub 2008/10/03. 18829008 10.1016/j.fertnstert.2008.08.025 PMC2818247

[52] Johansson K, Stephansson O, Neovius M. Outcomes of pregnancy after bariatric surgery. N Engl J Med. 2015;372(23):2267. PubMed PMID: 26039606. Epub 2015/06/04. 26039606 10.1056/NEJMc1503863

[53] Marceau P, Kaufman D, Biron S, Hould FS, Lebel S, Marceau S, et al. Outcome of pregnancies after biliopancreatic diversion. Obes Surg. 2004;14(3):318–24. PubMed PMID: 15072650. Epub 2004/04/10. 15072650 10.1381/096089204322917819

