# Supplementary material for: Menstrual and Reproductive Function Before and After Metabolic Bariatric Surgery
Source: Obes Surg. 2026 Aug 5;36(9):4535–43. doi: 10.1007/s11695-026-08852-6 (PMC13562210; doi:10.1007/s11695-026-08852-6)
Supplement: Supplementary file 1 [file 11695_2026_8852_MOESM1_ESM.docx]

**APPENDIX**

**Appendix Table 1. Characteristics of women with obesity recruited to a case control study of bariatric surgery.**

|  |  | Cases  N (%) | Controls  N (%) | P^1^ |
| --- | --- | --- | --- | --- |
| Ethnicity | White British/ European | 86 (87%) | 14 (78%) | 0.115 |
|  | Any other mixed background | 5 (5%) | 0 (0%) |  |
|  | Asian | 1 (1%) | 0 (0%) |  |
|  | Any other black background | 0 (0%) | 1 (5.5%) |  |
|  | Unknown | 7 (7%) | 3 (17%) |  |
| Deprivation | Mean decile (±SD) | 5.26 ±2.5 | 4.17±2.4 | 0.109† |
| Employment status | Full-time | 38 (38%) | 4 (22%) | 0.141 |
|  | Part-time^§^ | 21 (21%) | 6 (33%) |  |
|  | Looking after family/home | 9 (9%) | 6 (33%) |  |
|  | Not working due to sickness/disability | 9 (9%) | 1 (5.5%) |  |
|  | Unemployed | 2 (2%) | 1 (5.5%) |  |
|  | Student/ full-time education | 1 (1%) | 0 (0%) |  |
|  | Unknown | 19 (19%) | 0 (0%) |  |
| Educational attainment | University degree | 16 (16%) | 4 (22%) | 0.467 |
|  | Diploma | 6 (6%) | 1 (5.5%) |  |
|  | A-level or equivalent | 18 (18%) | 3 (17%) |  |
|  | GCSE or equivalent | 32 (32%) | 6 (33%) |  |
|  | Nil formal | 6 (6%) | 4 (22%) |  |
|  | Unknown | 21 (21%) | 0 (0%) |  |
| Smoking status | Never | 38 (38%) | 9 (50%) | 0.271 |
|  | Ex-smoker | 34 (34%) | 5 (28%) |  |
|  | Current smoker | 8 (8%) | 4 (22%) |  |
|  | Unknown | 19 (19%) | 0 (0) |  |
| Alcohol use | None | 58 (59%) | 10 (55%) | 0.169 |
|  | 1-14 units per week | 22 (22%) | 8 (44%) |  |
|  | 14+ units per week | 0 (0) | 0 (0%) |  |
|  | Unknown | 19 (19%) | 0 (0%) |  |
| Relationship status | Married/long-term partner | 56 () | 10 (55%) | 0.551 |
|  | Single | 19 () | 6 (33%) |  |
|  | Boyfriend/girlfriend | 4 () | 2 (11%) |  |
|  | Casual/dating | 1 (1%) | 0 (0%) |  |
|  | Unknown | 19 (19%) | 0 (0%) |  |

^1^p values derived from chi squared test, cases versus controls; ^†^p value derived from Mann-Whitney U-test, ^§^part-time working defined as <28hours/week

**APPENDIX: Reference ranges for AMH and testosterone**

**AMH levels by age and as a predictor of IVF success (provided by Wexham Park Hospital laboratory)**

| ^Age (years)^ | ^Reference Range (pmol/L)^ |
| --- | --- |
| ^20-24^ | ^10.9 – 71.0^ |
| ^25-29^ | ^8.6 – 64.6^ |
| ^30-34^ | ^5.1 – 54.2^ |
| ^35-39^ | ^2.9 – 49.7^ |
| ^40-44^ | ^0.4 – 31.7^ |
| ^45-50^ | ^0.1 – 12.8^ |

| ^The following AMH ranges can be used to predict response to IVF treatment, based on Antral Follicular Count as a surrogate marker^ | |
| --- | --- |
| ^Low responders^ | ^<4.9 pmol/L^ |
| ^Normal^ | 4.9 ^– 16.2 pmol/L^ |
| ^High responders^ | ^>16.2 pmol/L^ |

Testosterone (WTES)	Reference range for testosterone (mass spec):

Females (16 - 49 years): 0.3 - 1.7 nmol/L

Females (>= 50 years): 0.1 - 1.4 nmol/L

Males (18 - 49 years): 8.6 - 29.0 nmol/L

Males (>= 50 years): 6.7 - 25.7 nmol/L

Results are from Royal London Hospital,

Barts Health.
