# Supplementary material for: Menstrual and Reproductive Function Before and After Metabolic Bariatric Surgery
Source: Obes Surg. 2026 Aug 5;36(9):4535–43. doi: 10.1007/s11695-026-08852-6 (PMC13562210; doi:10.1007/s11695-026-08852-6)
Supplement: Supplementary file 2 [file 11695_2026_8852_MOESM2_ESM.docx]

**Reproductive Health after Metabolic surgery (RHAMS) Study Questionnaire**

Please complete the questionnaire as fully as you can. All your answers are confidential.

Thank you for taking part in this study.

**A little about you**

Are you

- A Student (please circle which you attend School/College/University)
- Working part-time (<28 hours/week)
- Working Full-time
- Looking after family/home
- Unemployed
- Not working because of sickness or disability

Who do you live with?

- Parents
- Partner/ husband
- Partner and (your/their) parents
- Other relatives or friends
- Alone
- Other (e.g. hall of residence, house provided with job etc…)

Are you in a relationship?

Yes/No

If yes, how would you describe it

- Married or Long-term partner
- Boyfriend or Girlfriend
- Casual relationship/ dating

Smoking status

- Current Smoker – how many per day
- Previously smoked – gave up	years ago
- Never smoker

Alcohol use

Average number of units per week

(approximate units in common drinks 25ml shot or vodka/gin = 1unit, glass of wine (175mls) = 2units, Pint of beer or cider = 2units, Alcopop bottle = 1 unit)

**About your reproductive health and history**

Do you have any problems with your periods?

Heavy	Yes/No

Painful	Yes/No

Irregular/absent	Yes/No

If yes: cycle length range	or number periods/year

(The number of days between first day of period until the first day of next period e.g 30-36days)

Other

Do you have acne?

Yes/No (please circle)

Do you suffer with excess body hair?

Yes/No (please circle)

**If yes:** where on your body do you have excess hair (please circle which looks most like you, if you are less hairy than number 1 then leave blank)

**Have you ever been pregnant?**

Yes/No (please circle)

If Yes please can you tell us a bit more about the pregnancies you have had

|  | Pregnancy 1 | Pregnancy 2 | Pregnancy 3 | Pregnancy 4 | Pregnancy 5 |
| --- | --- | --- | --- | --- | --- |
| Was it planned or unplanned |  |  |  |  |  |
| Conceived naturally or with fertility treatment  (Please say type of fertility treatment if you know) |  |  |  |  |  |
| What was the outcome of the pregnancy: Currently pregnant, Live birth, Miscarriage or Termination |  |  |  |  |  |

(If you have had more than five pregnancies please use the space at the end to tell us about them, thank you)

If No

Have you been trying to conceive?

For how long?

Have you undergone fertility treatment? What?

What best describes your pregnancy plans?

- Do not want to get pregnant
- Not sure
- want to get pregnant as soon as possible
- want to get pregnant in the next	(please say how soon you want to get pregnant)
- Want to get pregnant, but no planned timeframe

What is your current method of contraception?

- None – not having sex
- None – having sex but don’t expect to get pregnant
- None – trying to get pregnant
- Condoms
- Pills (oral contraceptive)
  - If you know is it a combined method or progesterone only (please circle)
  - Name?
- Contraceptive injection (Depo)
- Contraceptive implant
- Coil – Copper or Mirena or Jaydess (please circle if you know which type)
- Other

Are you happy with your current contraception?

Yes/ No (please circle)

If no, why not

**Thank you for your time**
