# Supplementary material for: Menstrual and Reproductive Function Before and After Metabolic Bariatric Surgery
Source: Obes Surg. 2026 Aug 5;36(9):4535–43. doi: 10.1007/s11695-026-08852-6 (PMC13562210; doi:10.1007/s11695-026-08852-6)
Supplement: Supplementary file 3 [file 11695_2026_8852_MOESM3_ESM.docx]

**Reproductive Health after Metabolic surgery (RHAMS) Study Questionnaire**

(Modified from the BOSS-42 Questionnaire, Tayyem et al 2014)

Please complete the questionnaire as fully as you can, boxes follow each question if you want to write more. If answering the questions makes you anxious or upset please get in touch with the research team or your GP for further advice. If when you finish the questionnaire there is anything else you want to say please use the space at the end.

All your answers are confidential, the only reason for breaking confidentiality is if you said you had thoughts of hurting yourself or others.

Thank you for taking part in the study.

**A little about you**

Smoking status

- Current Smoker – how many per day
- Previously smoked – gave up	years ago
- Never smoker

Education level

Age left school

- No formal qualification
- GCSE or equivalent
- A –Level or equivalent
- University degree
- Other

Are you

- A Student (please circle which you attend School/College/University)
- Working part-time (<28 hours/week)
- Working Full-time
- Looking after family/home
- Unemployed
- Not working because of sickness or disability

Who do you live with?

- Parents
- Partner/ husband
- Partner and (your/their) parents
- Other relatives or friends
- Alone
- Other (e.g. hall of residence, house provided with job etc…)

Are you in a relationship?

Yes/No

If yes, how would you describe it

- Married or Long-term partner
- Boyfriend or Girlfriend
- Casual relationship/ dating

Has anyone else in your family had bariatric surgery?

Yes/ No, If yes who

What is your main reason for having bariatric surgery?

- Health/ Medical
- Your doctor recommended it
- Family or friends suggested it
- Remarks by others
- Sexual/ relationship
- To help get pregnant
- How you look
- Other

**About your diet and any supplements you take**

To try and understand your diet, could you please tell us what you have eaten over the last two days?

Please give as much detail as you can on what you ate, how it was prepared and the amounts eaten. You could use weights from labels, spoonfuls or handfuls to describe the amounts eaten. Please carry on overleaf if you need more space.

|  | Today (up until now) | Yesterday |
| --- | --- | --- |
| Breakfast |  |  |
| Mid morning |  |  |
| Lunch |  |  |
| Mid afternoon |  |  |
| Dinner |  |  |
| Evening |  |  |
| Snacks |  |  |
| Drinks |  |  |

What vitamin/ mineral supplements have you been advised to take and do you take them regularly?

| Vitamin/ mineral | Dose and how often (if you know it) | Are you currently taking  (yes/no) | If not taking regularly, why is this (e.g. cost/ side effects / forgetting/ don’t feel you need it etc) |
| --- | --- | --- | --- |
|  |  |  |  |
|  |  |  |  |
|  |  |  |  |
|  |  |  |  |
|  |  |  |  |
|  |  |  |  |
|  |  |  |  |

**About your reproductive health and history**

How old were you when you had your first period?

Have you ever been diagnosed with polycystic ovarian syndrome

Yes/No

If yes by who?

Do you have any problems with your periods?

Heavy	Yes/No

Painful	Yes/No

Irregular/absent	Yes/No

If yes: cycle length range	or number periods/year

(The number of days between first day of period until the first day of next period e.g 30-36days)

Other

Do you have acne?

Yes/No (please circle)

Do you suffer with excess body hair?

Yes/No (please circle)

**If yes:** where on your body do you have excess hair (please circle which looks most like you, if you are less hairy than number 1 then leave blank)

**Have you ever been pregnant?**

Yes/No (please circle)

If Yes please can you tell us a bit more about the pregnancies you have had

|  | Pregnancy 1 | Pregnancy 2 | Pregnancy 3 | Pregnancy 4 | Pregnancy 5 |
| --- | --- | --- | --- | --- | --- |
| Was it planned or unplanned |  |  |  |  |  |
| Conceived naturally or with fertility treatment  (Please say type of fertility treatment if you know) |  |  |  |  |  |
| What was the outcome of the pregnancy: Currently pregnant, Live birth, Miscarriage or Termination |  |  |  |  |  |

(If you have had more than five pregnancies please use the space at the end to tell us about them, thank you)

If No

Have you been trying to conceive?

For how long?

Have you undergone fertility treatment? What?

What best describes your pregnancy plans?

- Do not want to get pregnant
- Not sure
- want to get pregnant as soon as possible
- want to get pregnant in the next	(please say how soon you want to get pregnant)
- Want to get pregnant, but no planned timeframe

What is your current method of contraception?

- None – not having sex
- None – having sex but don’t expect to get pregnant
- None – trying to get pregnant
- Condoms
- Pills (oral contraceptive)
  - If you know is it a combined method or progesterone only (please circle)
  - Name?
- Contraceptive injection (Depo)
- Contraceptive implant
- Coil – Copper or Mirena or Jaydess (please circle if you know which type)
- Other

Are you happy with your current contraception?

Yes/ No (please circle)

If no, why not

**Some questions about how happy you are with your body, your support networks and general well-being**

| **In the last FOUR weeks how satisfied have you been with** | | | | | |
| --- | --- | --- | --- | --- | --- |
| Your current weight? | Very Dissatisfied | Dissatisfied | Neutral | Satisfied | Very Satisfied |
| The way your body looks? | Very Dissatisfied | Dissatisfied | Neutral | Satisfied | Very Satisfied |
| The support you get from your *family*? | Very Dissatisfied | Dissatisfied | Neutral | Satisfied | Very Satisfied |
| The support you get from friends? | Very Dissatisfied | Dissatisfied | Neutral | Satisfied | Very Satisfied |
| Your sex life | Very Dissatisfied | Dissatisfied | Neutral | Satisfied | Very Satisfied |
| Your life in general | Very Dissatisfied | Dissatisfied | Neutral | Satisfied | Very Satisfied |

| **In the last FOUR weeks did you feel** | | | | | |
| --- | --- | --- | --- | --- | --- |
| Your family understood what you were going through? | Strongly Disagree | Disagree | Neither | Agree | Strongly Agree |
| Your friends were sympathetic and encouraging? | Strongly Disagree | Disagree | Neither | Agree | Strongly Agree |
| Happy with your physical appearance? | Strongly Disagree | Disagree | Neither | Agree | Strongly Agree |
| Sexually attractive? | Strongly Disagree | Disagree | Neither | Agree | Strongly Agree |
| Content with your life? | Strongly Disagree | Disagree | Neither | Agree | Strongly Agree |
| More energised or invigorated? | Strongly Disagree | Disagree | Neither | Agree | Strongly Agree |

| **During the last FOUR weeks would you describe being** | | | | | |
| --- | --- | --- | --- | --- | --- |
| Comfortable with your body image? | Strongly Disagree | Disagree | Neither | Agree | Strongly Agree |
| More physically active? | Strongly Disagree | Disagree | Neither | Agree | Strongly Agree |
| More sociable with friends? | Strongly Disagree | Disagree | Neither | Agree | Strongly Agree |
| More satisfied with life? | Strongly Disagree | Disagree | Neither | Agree | Strongly Agree |
| Happy with your health? | Strongly Disagree | Disagree | Neither | Agree | Strongly Agree |
| Supported more by your family? | Strongly Disagree | Disagree | Neither | Agree | Strongly Agree |

| **Do you feel your weight limits you in:** | | | | | |
| --- | --- | --- | --- | --- | --- |
| Social activities such as visiting friends? | Very Severe Limitation | Severe Limitation | Moderate Limitation | Little Limitation | No Limitation at all |
| Picking up objects? | Very Severe Limitation | Severe Limitation | Moderate Limitation | Little Limitation | No Limitation at all |
| Carrying out your daily activities? | Very Severe Limitation | Severe Limitation | Moderate Limitation | Little Limitation | No Limitation at all |
| Enjoying your free time? | Very Severe Limitation | Severe Limitation | Moderate Limitation | Little Limitation | No Limitation at all |
| Your ability to get a new job? | Very Severe Limitation | Severe Limitation | Moderate Limitation | Little Limitation | No Limitation at all |

| **Because of your current weight/ appearance do you think it is likely that:** | | | | | |
| --- | --- | --- | --- | --- | --- |
| You will have fewer friends? | Definitely False | Mostly False | Not Sure | Mostly True | Definitely True |
| Your health will get worse? | Definitely False | Mostly False | Not Sure | Mostly True | Definitely True |
| You will be embarrassed during sex? | Definitely False | Mostly False | Not Sure | Mostly True | Definitely True |
| Your family will be less supportive? | Definitely False | Mostly False | Not Sure | Mostly True | Definitely True |
| You will no longer be able to enjoy things you used to? | Definitely False | Mostly False | Not Sure | Mostly True | Definitely True |
| Your life may end prematurely? | Definitely False | Mostly False | Not Sure | Mostly True | Definitely True |
| Strangers will be more critical or judgemental? | Definitely False | Mostly False | Not Sure | Mostly True | Definitely True |

| **How would you best describe your approach to food and eating** | | | | | |
| --- | --- | --- | --- | --- | --- |
| You prefer more health food options | Strongly Disagree | Disagree | Neither | Agree | Strongly Agree |
| You eat until you feel uncomfortably full | Strongly Disagree | Disagree | Neither | Agree | Strongly Agree |
| Eating is a pleasurable experience | Strongly Disagree | Disagree | Neither | Agree | Strongly Agree |
| You find it difficult to stop eating once you’ve started | Strongly Disagree | Disagree | Neither | Agree | Strongly Agree |
| Food choices are based on foods you enjoy | Strongly Disagree | Disagree | Neither | Agree | Strongly Agree |
| You eat more than you body needs | Strongly Disagree | Disagree | Neither | Agree | Strongly Agree |

| **In the last FOUR weeks, to what extent** | | | | | |
| --- | --- | --- | --- | --- | --- |
| Did you like the shape of your body? | Much less | Less | Neither | More | Much more |
| Were you happy with you sex drive/urge? | Much less | Less | Neither | More | Much more |
| Were your family understanding and caring? | Much less | Less | Neither | More | Much more |
| Were your friends there for you? | Much less | Less | Neither | More | Much more |
| Were you at ease with yourself? | Much less | Less | Neither | More | Much more |

| I**n the last FOUR weeks, did you experience:** | | | | | |
| --- | --- | --- | --- | --- | --- |
| Having low sexual desire? | Definitely False | Mostly False | Not Sure | Mostly True | Definitely True |
| Hating the appearance of your body? | Definitely False | Mostly False | Not Sure | Mostly True | Definitely True |
| Being unsociable/withdrawn? | Definitely False | Mostly False | Not Sure | Mostly True | Definitely True |
| Physical fatigue/tiredness | Definitely False | Mostly False | Not Sure | Mostly True | Definitely True |

| **In the last FOUR weeks have you suffered from**: | | | | | |
| --- | --- | --- | --- | --- | --- |
| Back pain? | Not at all | Slightly | Moderately | A great deal | Very much |
| Shortness of breath? | Not at all | Slightly | Moderately | A great deal | Very much |
| Snoring? | Not at all | Slightly | Moderately | A great deal | Very much |
| Painful joints? | Not at all | Slightly | Moderately | A great deal | Very much |
| Difficulties with sleeping? | Not at all | Slightly | Moderately | A great deal | Very much |
| Difficulties with transport? | Not at all | Slightly | Moderately | A great deal | Very much |

Final thoughts/ comments

please use this space to either write more in answer to any of the questions or anything else you would like to share about your motivations to have surgery.

**Thank you for your time**
